# The Asylum Tragedy

**Published:** 1892-01

**Authors:** 


### EDITORIAL DEPARTMENT.

F. E. DANIEL, M. D., EDITOR AND PUBLISHER.

THIS JOURNAL, by unanimous vote of the members, was made the Official Organ of the Austin District Medical Society.

COLLABORATORS:

*Dr. R. M. Swearingen, Austin.*
*Prof. B. E. Hadra, M. D., Galveston.*
*Prof. Geo. Cupples, M. D., San Antonio.*
*Dr. T. C. Osborn, Cleburne, Texas.*
*Dr E. J. Doering, Chicago.*
*Dr. E. J. Beall, Fort Worth.*
*Dr Odo Betz, Germany.*
*Prof. W. B. Rogers, M. D., Memphis.*
*Dr. J. W. McLaughlin, Austin.*
*Dr. T. J. Tyner, Austin.*
*Prof. J. F. Y. Paine, M. D., Galveston.*
*Dr. R. H. L. Bibb, Mexico.*
*Dr. T. J. Bennett. Austin.*
*Dr. Bat Smith, Wharton.*
*Dr. E. Meierhof, New York.*
*L. H. Luce, M. D., West Tisbury, Mass.*

### THE ASYLUM TRAGEDY.

Nothing could be more startling, than the announcement that the Superintendent of the State Lunatic Asylum had been shot down by a lunatic, with a double-barreled shot gun. The question would naturally be asked, how did the patient get the gun? How did he happen to be at large?

These questions are the more pertinent, from the fact that the man was known to be subject to fits of insanity, and in that state was, of course, irresponsible, and liable to do anything.

It would seem at first glance that there had been gross carelessness somewhere, or that "somebody had blundered."

It is human nature to throw the blame for all such occurrences on some one, and in this instance it would be interesting to know where, if any. the blame attaches.

Dr. Reeves was an accomplished physician, and an elegant gentleman. Under his administration everything was working smoothly and satisfactorily, and his career was certainly most promising. He was a general favorite, and his loss is deeply deplored. But he had had no experience, previously, with lunatics, and there are those who say that if he had had, he would either not have permitted Purnell to go at large or, knowing that he was subject to insane, and therefore dangerous periods, he would never have spoken authoritatively to him,—this class of patients being, as is well known, most sensitive, and prone to take offense.

The story of the killing is published elsewhere in this issue, substantially as it appeared in the daily papers, and the man's history, taken from the record book at the asylum, is appended. It appears that Purnell took offense at Dr. Reeves, because he would not allow him to sit and read in the office of the asylum, and spoke to him once or twice about it, rather sharply at last. This is assigned as the direct motive that led to the killing.

In the opinion of many, THE JOURNAL amongst them, the blame lies deeper. It may have been the outcome of a condition of things at present unavoidable. It seems that Purnell was discharged as "restored." He had been thrice "restored," previously, and on appearance of symptoms of a return of his malady, had been readmitted into the asylum. The last time, Nov. 1, '91, he was discharged *against his wishes.*

With a record like the above, why was he ever allowed to go at large? Why turned out against his will? Why was he not kept, as he should have been, under constant surveillance, and taken care of on the first appearance of a return of his trouble? *Because there is not room enough in the asylums of Texas to properly care for her afflicted*, and Henry Purnell was discharged as "restored," with the almost absolute certainty of a relapse.—warranted by his past history, as are many others—to make room for more urgent cases; and still, the jails and the workhouses all over the State, as well as the two magnificent "palaces" erected for the insane, are full of patients. Henry was only periodically insane; most lunatics are; are the periodically insane discharged to make room for others? Surely not; then, why was Purnell made an exception? Why, when he expressed fears to Mr. Thornton that he would "hurt somebody," did his friends not return him to the asylum? Surely it is the fault of somebody that a condition of things exists in which it is possible for an insane man—known to be subject to spells of insanity—to get a gun and kill the Superintendent of the asylum or any other person. We must assume that Dr. Reeves was conscientious in doing his duty, as he understood it; otherwise he would not have discharged Purnell. It was an error of judgment, or—it may have been a necessity.

Nor can we blame the legislature. They appreciate the situation, and have a humane regard for the wants and necessities of the growing class of unfortunates. Large appropriations have been made for them; costly, magnificent buildings have been erected, and recently another splendid asylum has been erected at San Antonio, not yet occupied, and still, all are full to overflowing—and many unfortunates, as stated, are in jail or elsewhere, and cannot be cared for; hence the *necessity* of *discharging* those who are really not fit to be discharged.

The trouble is, medical men are not consulted, as a general thing, by the legislators, when they come to legislating on this subject, or any other, on which physicians are better informed than they. They do not use the appropriations judiciously—they are ample, if used with better judgment.

Instead of building any more costly "palaces" for the insane, as Dr. White calls them, where convalescents see too much brick and mortar, and not enough "nature;" let the State adopt Dr. White's suggestion* and buy one or more large tracts of land for asylum purposes. On these build cottages for the convalescents, partially restored or chronic incurables, who are able to work, and give them suitable employment, such as farming, gardening, or work at some light trade, such as is followed in other institutions. This would make room for the severe cases in the grand asylum building, and as the convalescents or improved class increased, they could be transferred to these cottages and still make room. This would necessitate only the occasional building of an additional cottage, instead of, at long intervals,—during which such a state of affairs as exists at present, could come about,—building another hundred and fifty-thousand dollar *pile*. These improved, or chronics, or convalescents, while having the benefit of wholesome work, and out-door scenery and air,—would still be under observation, in case of a relapse or outbreak, and could be promptly cared for. Such farm or settlement could also be made self-sustaining, if not a source of revenue.

It is an injustice to society, and a cruelty to the patient himself, that such an one as Purnell should be thrown on his own resources, during his lucid or well moments, especially, if he be liable, as Purnell was, to sudden seizure at any time. As well abandon an epileptic to himself, to fall in the fire, or down the steps, the first time he has a fit.

---

*"What to Do With the Insane," by F. S. White, M. D., late 1st Asst. Phys. State Lunatic Asylum, Terrell (and, since the above was in type, appointed Superintendent of the Asylum at Austin, to fill the vacancy caused by Dr. Reeves' death), in DANIEL'S TEXAS MEDICAL JOURNAL, Sept, 1891.

Let the legislature take this costly lesson to heart, and provide against a repetition of it. Let them consider Dr. White's proposition; it appears to THE JOURNAL a rational solution of the problem what to do with the excess of insane over accommodations, and the only one compatible with public safety and the welfare of the afflicted mentally.

